# Counting the costs of drinking alcohol during pregnancy

**DOI:** 10.2471/BLT.17.030517

**Published:** 2017-05-01

## Abstract

Researchers are starting to shed light on the true extent of alcohol consumption during pregnancy. Svetlana Popova talks to Fiona Fleck.

Q: What is fetal alcohol syndrome (FAS) and how much do we know about it today? 

A: Alcohol is poisonous to the developing fetus throughout the entire nine months of gestation. When a mother-to-be consumes alcohol, it goes directly to the fetus through her blood stream. These children may be born with fetal alcohol spectrum disorder (FASD), which is an umbrella term that covers all alcohol-related diagnoses, of which fetal alcohol syndrome (FAS) is the most severe and visibly identifiable form. FASD is associated with a wide range of physical, behavioural and learning problems including growth impairments, facial abnormalities, problems with brain function and developmental delays. Recently our team identified more than 400 conditions that co-occur in individuals with FASD, spanning 18 of the 22 chapters of the *International statistical classification of diseases and related health problems*. Many of these conditions occur more often among people with FASD than in the general population, although a causal link has only been made with some of these conditions. 

Q: When did you first become aware of these disorders? 

A: When I studied psychiatry as a medical student, I remember a few lines in our textbook saying that alcoholic mothers may deliver children with birth defects and other malformations. That was all there was in the curricula for medical students in the former Soviet Union. Physicians were not trained to recognize FASD and this is still the situation in many countries today. 

Q: How did research start in this area? 

A: FAS was first described in the French medical literature by Paul Lemoine and colleagues in a 1968 study of children of alcohol-dependent parents. Five years later, Ken Jones and David Smith published a paper in the *Lancet* on the association between alcohol abuse and morphological signs, and provided diagnostic criteria for this condition. 

Q: How did you start researching these disorders? 

A: The Centre for Addiction and Mental Health, where I work, is a WHO collaborating centre and I started researching FASD in 2009, when WHO asked our team to collaborate on developing a method that could be used to estimate the prevalence of FAS and FASD in all countries. The method was presented to and discussed with researchers from around the world at the first WHO global expert Meeting on Alcohol, Health and Development in Sweden in 2009. It was then refined with technical input from the National Institute on Alcohol Abuse and Alcoholism in the USA and discussed at a WHO planning meeting of principal investigators from more than 15 countries, organized alongside the first European conference on FASD in the Netherlands in 2010. I found that this field was severely under researched and since then I have become dedicated to research in this area.

*Q. Can you tell us about your study in *The**Lancet Global Health* on the global prevalence of maternal drinking and FAS published in January?*

A. We wanted to draw the attention of health-care practitioners, health authorities and policy-makers to the problem of maternal alcohol consumption and FASD. As epidemiologists we know how important it is to determine the prevalence of a disorder in order to set priorities for public health policy, funding for public health initiatives and health-care planning. Most countries did not have prevalence data at a population level for alcohol use during pregnancy or FAS, so this study was urgently needed.

Q: What were the key findings?

A: We estimated that one out of 67 women who consume alcohol during pregnancy will deliver a child with FAS, which translates to about 119 000 such children born globally every year. We knew before the study that not every woman who drinks alcohol during pregnancy will deliver a child with FAS, because women drink different amounts and every mother and every fetus has a different ability to metabolize alcohol, and there are many other factors that may influence their vulnerability. Before our study, most governments had no idea how many pregnant women drink alcohol and how many children are born with FAS in their countries. Now, countries can use these data to help children with FAS and to prevent future cases. 

Q. Which countries had the highest and lowest estimates? 

A: The five countries with the highest prevalence of alcohol use during pregnancy were Ireland (about 60%), Belarus (47%), Denmark (46%), the United Kingdom of Great Britain and Northern Ireland (41%) and the Russian Federation (37%) – all of these in the WHO European Region. The lowest prevalence was observed in the WHO Eastern Mediterranean Region, where most people – including pregnant women of course – abstain from alcohol due to religious beliefs. Overall, we found that alcohol use during pregnancy is common in many countries and that FAS is a relatively prevalent birth defect.

Q: How common is FASD compared with other birth defects? 

A: We recently found that the**prevalence of FASD is elevated and exceeds 1% in many countries, these data are not published yet. This suggests that in some countries the prevalence of FASD may be higher than the prevalence of some common – and more generally known – birth defects such as anencephaly, Down syndrome, spina bifida and trisomy 18.

“These figures should not further stigmatize mothers of children with FASD, but rather help to prioritize funding and support for these families.”

Q: What are the economic costs of FASD? 

A: While we were working on the Global FASD prevalence project, the Public Health Agency of Canada asked our team to estimate the economic cost of FASD in Canada. We found huge costs in terms of law enforcement, the provision of social services and special education, as well as productivity losses due to morbidity and premature mortality. Even with the assumption that only 1% of the Canadian population have FASD – about 355 000 people – the annual cost was estimated at 1.8 billion Canadian Dollars (US$ 1.35 billion) per year in Canada. These estimates should not further stigmatize the mothers of children with FASD, but rather help to prioritize funding and support for these families. These figures are the minimum costs associated with FASD in Canada and don’t include the cost incurred to individuals themselves and their families.

Q: If this is such a common condition, why is there so little awareness of it?

A: Even for the health sector, FASD is a relatively new condition. We’ve made progress in understanding how alcohol damages the fetus, but this has not yet translated into better public awareness of the risks. We estimate that globally, on average, one in 10 women consumes alcohol during pregnancy and 20% of these women binge drink, which means they consume four or more alcoholic drinks per single occasion. Binge drinking is the direct cause of FAS or FASD. These findings are alarming because half of the pregnancies in developed countries and over 80% in developing countries are unplanned. That means that many women don’t realize they are pregnant during the early stages and that they continue drinking when pregnant. 

Q: Why are women drinking so much alcohol today? 

A: Men still drink more alcohol than women, but the epidemiology of alcohol use appears to be changing and the gap between male and female patterns of alcohol use is closing, especially at younger ages. Women’s alcohol consumption has been increasing in line with economic development and changing gender roles, but other factors include marketing directed towards women, increased availability and accessibility of alcoholic beverages and increased social acceptability of women drinking alcohol.

Q: How can the global public health community use your global burden of disease study to prevent fetal alcohol disorders in their countries? 

A: We provided data on 187 countries. At a recent international conference on FASD in Vancouver many people from different countries thanked our team for providing them with the data and evidence that they can use as the basis for prevention measures. They said that before, when they told their governments that FASD is a serious disabling, prevalent and costly condition, government officials dismissed them and some didn’t even believe that FASD existed. 

Q: Can you tell us about the new WHO study your centre is working on with the National Institute on Alcohol Abuse and Alcoholism? 

A: WHO initiated an International Collaborative Research Project on Child Development and Prenatal Risk factors with a focus on FASD to help gain a better understanding of the prevalence, severity and impact of FASD. This research is designed to inform policies and programmes to reduce the harmful use of alcohol among women of childbearing age and to prevent alcohol consumption among pregnant women. Multidisciplinary teams of experts from different institutions around the world are collaborating on this project. We are estimating the prevalence of FASD by screening children aged 7–9 years from different populations in Belarus, Canada, Moldova, Namibia, the Seychelles and Ukraine.

“More should be done to inform women about the harmful effects of drinking alcohol during pregnancy.”

Q: What is your hope for the future? 

A: Alcohol consumption during pregnancy should be recognized as a serious public health problem and more effective prevention strategies targeting alcohol use before and during pregnancy are needed worldwide. More should be done to inform women about the harmful effects of drinking alcohol during pregnancy. Where appropriate, universal screening of pregnant women and women of childbearing age for alcohol use could be established. We need an FASD surveillance system to monitor the incidence and prevalence of FASD globally. We are all responsible for the prevention of FASD: partners, families, friends and communities should all help and support women during pregnancy. People with FASD – a preventable, but invisible disability - should not be forgotten by our societies. 

